# The Neurotoxicity of Vesicles Secreted by ALS Patient Myotubes Is Specific to Exosome-Like and Not Larger Subtypes

**DOI:** 10.3390/cells11050845

**Published:** 2022-03-01

**Authors:** Ekene Anakor, Vanessa Milla, Owen Connolly, Cecile Martinat, Pierre Francois Pradat, Julie Dumonceaux, William Duddy, Stephanie Duguez

**Affiliations:** 1 Personalised Medicine Centre, Biomedical Sciences Research Institute, Ulster University, Derry-Londonderry BT47 6SB, UK; Anakor-E@ulster.ac.uk (E.A.); milla-v@ulster.ac.uk (V.M.); Connolly-O4@ulster.ac.uk (O.C.); pierre-francois.pradat@aphp.fr (P.F.P.); j.dumonceaux@ucl.ac.uk (J.D.); w.duddy@ulster.ac.uk (W.D.); 2 I-STEM, INSERM/UEVE UMR 861, AFM, 91100 Corbeil-Essones, France; CMARTINAT@istem.fr; 3 Laboratoire d’Imagerie Biomédicale, Sorbonne Université, CNRS, INSERM, 75013 Paris, France; 4 Centre référent SLA, Département de Neurologie, Hôpital Pitié-Salpêtrière, APHP, 75013 Paris, France; 5 NIHR Biomedical Research Centre, Great Ormond Street Institute of Child Health, Great Ormond Street Hospital NHS Trust, University College London, London WC1N 1EH, UK

**Keywords:** exosomes, ectosomes, cell–cell communication, motor neurone diseases

## Abstract

Extracellular vesicles can mediate communication between tissues, affecting the physiological conditions of recipient cells. They are increasingly investigated in Amyotrophic Lateral Sclerosis, the most common form of Motor Neurone Disease, as transporters of misfolded proteins including SOD1, FUS, TDP43, or other neurotoxic elements, such as the dipeptide repeats resulting from *C9orf72* expansions. EVs are classified based on their biogenesis and size and can be separated by differential centrifugation. They include exosomes, released by the fusion of multivesicular bodies with the plasma membrane, and ectosomes, also known as microvesicles or microparticles, resulting from budding or pinching of the plasma membrane. In the current study, EVs were obtained from the myotube cell culture medium of ALS patients or healthy controls. EVs of two different sizes, separating at 20,000 or 100,000 g, were then compared in terms of their effects on recipient motor neurons, astrocytes, and myotubes. Compared to untreated cells, the smaller, exosome-like vesicles of ALS patients reduced the survival of motor neurons by 31% and of myotubes by 18%, decreased neurite length and branching, and increased the proportion of stellate astrocytes, whereas neither those of healthy subjects, nor larger EVs of ALS or healthy subjects, had such effects.

## 1. Introduction

Cell–cell communication occurs via a variety of mechanisms including secretion of soluble factors, direct contact via tunnelling nanotubes and cytonemes, as well as extracellular vesicles [1]. Extracellular vesicles (EVs) have garnered great interest owing to their ability to mediate near as well as distant communication within and between different cell types and tissues, impacting the pathological and physiological conditions of the targeted cells, as observed in metastatic cancer and in neurodegenerative diseases [2,3,4]. EVs are constitutively secreted [5,6] and can be classified based on their biogenesis and size as: exosomes, ectosomes, or apoptotic bodies. Exosomes are formed when multivesicular bodies (MVBs) containing intraluminal vesicles fuse with the plasma membrane, releasing vesicles that typically measure between 50 and 200 nm [7,8,9]. Ectosomes, also known as microvesicles or microparticles, result from the direct budding or pinching off the plasma membrane in response to increased calcium influx, representing a disruption in plasma membrane phospholipid architecture [10], and usually having a size distribution of 100–1000 nm [11]. Finally, apoptotic bodies are released from apoptotic or dying cells, containing fragments with a wide variety of cellular components, and measuring between 500 nm and 2 μm [12]. Of these, exosomes and ectosomes are known to carry functional proteins and RNAs to recipient cells [9,13,14,15].

The skeletal muscle, which represents 40% of total body mass [16], is known to release myokines and extracellular vesicles (exosomes and microparticles) [13,17,18,19] that have roles in the myogenic program [14,20] and in tissue maintenance and adaptation [21,22]. Muscle vesicle secretion is stimulated under various conditions, including vigorous exercise, hypoxia, inflammation and neurodegenerative diseases [23,24,25]. These vesicles contain functional proteins and RNA cargoes [13], and can have an impact on myogenesis and muscle regeneration [26,27,28], neurite branching and outgrowth, as well as on motor neuron survival and regeneration accuracy [29,30]. In pathological conditions, EVs secreted by muscle can impact distal organs such as the pancreas or liver [31].

Amyotrophic Lateral Sclerosis (ALS) is a disease with an adult onset characterized by the degeneration of upper and lower motor neurons, affecting between 1 and 2.6 per 100,000 people per year [32]. While many mechanisms are advocated to be responsible for the disease [33], the role of extracellular vesicles in the progression of ALS is increasingly investigated [9,34,35,36]. Exosomes originating from the astrocytes of ALS murine models [36,37], or from ALS patient iPSC-derived astrocytes [38] and motor neurons [36], have been implicated in pathogenesis [37,39,40,41], carrying misfolded proteins such as SOD1, FUS, TDP43, or toxic elements such as the dipeptide repeats (DPRs) resulting from *C9orf72* expansions [36,37,40,42,43]. In alignment with these studies, we recently observed that the skeletal muscle cells of ALS patients secrete exosome-like vesicles (MuVs) that are toxic towards motor neurons [44].

In this context, we wanted to determine whether MuVs and large muscle vesicles (lmEVs—extracted at 20,000 g) would be similarly taken up by, and exert similar effects on, motor neurons, astrocytes and muscle cells.

## 2. Materials and Methods

### 2.1. Culture of Primary Human Myoblasts and Differentiation into Myotubes

Primary human myoblasts were obtained from human deltoid muscle biopsies from a previous study (*n* = 7 ALS and *n* = 7 healthy) [44]. The protocol (NCT01984957) was approved by the local Ethical Committee and all subjects signed an informed consent in accordance with institutional guidelines. ALS gene mutations had been assessed previously [44], and included testing for the *C9orf72* hexanucleotide repeat expansion, *ATXN2* repeat length, and the coding regions of *SOD1*, *TARDBP*, *FUS*, *UBQLN2* and *TBK1*. Subject characteristics are given in Table 1.

CD56-sorted myoblasts were expanded in 0.22 µm filtered proliferating medium containing DMEM/M199 (Thermo Scientific) supplemented with 20% FBS, 25 µg·mL^−1^ Fetuin, 0.5 ng·mL^−1^ basic fibroblast growth factor (bFGF), 5 ng/mL epidermal growth factor (EGF), 5 µg·mL^−1^ insulin and incubated at 5% CO_2_, 37 °C. Enrichment in myoblasts was confirmed for all cultures, with over 90% of the cells positive for desmin. The healthy myoblasts differentiated into myotubes from subject F aged 50–59 were used to test the uptake and effect of EVs.

For extraction of extracellular vesicles, myoblasts were plated at a density of 33,400 cells·cm^−2^ as described in [45] (7.5 × 10^6^ myoblasts in 225 cm^2^ flasks (Falcon™)) for 24 h before washing six times with supplement-free DMEM. The cells were then differentiated in DMEM to form myotubes, and conditioned culture medium was collected at 72 h. All cell cultures were regularly checked for mycoplasma infection.

### 2.2. Isolation of Extracellular Vesicles from Conditioned Media

EVs were extracted as previously described, at myoblast cell division counts of less than 20 in order to avoid potential artefacts due to pre-senescence [13,44,45]. Briefly, the conditioned cell culture media from differentiated myoblasts was centrifuged at 260 g for 10 min and the resulting supernatant centrifuged at 4000 g for 20 min. The lmEVs were then pelleted by ultracentrifugation at 20,000 g for 70 min at 4 °C. The MuVs were extracted and washed from the remaining supernatant as previously described [45]. The lmEVs were washed thrice with PBS (20,000 g for 70 min at 4 °C) and resuspended in PBS for functional studies or lysed in NuPage/RIPA buffer for Western blot analysis. Similarly, MuVs were resuspended in PBS for functional studies or lysed in NuPAGe/RIPA for Western blot experiments.

### 2.3. Nanoparticle-Tracking Analysis (NTA)

Size-distribution analysis and quantification of EV preparations were performed on a NanoSight LM10 (Malvern Panalytical) equipped with fast video capture and particle tracking software. Purified vesicles from differentiated myoblasts were diluted into 1 mL of PBS. Five videos of 1 min duration were recorded for each sample, with a frame rate of 25 frames per second and analyzed using NanoSight software (NTA 3.4 Build 3.4.003). The camera level was set at 11, the temperature was 23.4 °C and the viscosity was 0.920–0.922 cP. The data on the sizes of EVs are expressed as the calculated means ± SD of size distribution.

### 2.4. Protein Quantification for MuVs and lmEVs

Muscle EVs were lysed by adding 50 μL of RIPA lysis buffer containing 1X Halt™ protease inhibitor cocktail (Thermo Scientific™, Waltham, MA, USA). After 10 min of incubation at 4 °C, the samples were centrifuged at 10,000 g for 10 min at 4 °C. The supernatants were collected, and protein concentration was spectrophotometrically measured at 570 nm using the Pierce™ BCA Protein Assay kit (Thermo Scientific™).

### 2.5. Lipid Quantification for MuVs and lmEVs

The total lipid concentration of EVs in PBS was determined according to the method of [46]. Briefly, chloroform was added to the lipid standard (olive oil prepared at a stock concentration of 2 mg·mL^−1^). Thereafter, 750 µL concentrated sulphuric acid was added to the standard and to the MuV and lmEV samples. After vortexing, standards and samples were incubated at 90 °C for 20 min. The absorbance was measured spectrophotometrically at 540 nm after adding phosphovanillin acid (0.2 mg·mL^−1^ vanillin in 17% phosphoric acid).

### 2.6. Western Blotting for MuVs and lmEVs

EV protein extracts were prepared under non-reducing or reducing conditions as previously described [45], heated at 70 °C for 10 min and electrophoresed on a 4–12% NuPAGE^®^ Bis-Tris gel (Thermo Fisher Scientific, Invitrogen) in MOPS-SDS Running buffer (Life Technologies™, Prague, Czech Republic) for 60 min at 200 V. The polyacrylamide gel was then incubated in 20% ethanol for 10 min and the separated proteins transferred onto a polyvinylidene fluoride (PVDF) membrane using the iBlot2 Dry Blotting System. The iBind™ Flex Western Device (Life Technologies™) was used for immunoblotting and performed according to manufacturer’s recommendation. The membranes were probed with primary antibodies for CD63 (10628D, Invitrogen, 1:1000), CD81 (10630D, Invitrogen, 1:1000), CD82 (PA5–79006, Invitrogen, 1:1000), Annexin A1 (A305-234A, Bethyl Laboratories, 1:1000), ARF6 (A235-238A, Bethyl Laboratories, 1:1000), alpha skeletal-Actin (PA5-117294, Invitrogen, 1:1000) as well as goat anti-mouse, anti-rabbit or sheep anti-mouse secondary antibodies conjugated with HRP (Thermo Fisher Scientific 1:8000; 1:10,000 and 1:8000, respectively). Subsequently, the membranes were incubated with Amersham ECL Prime Western Blotting Detection Reagent for 2 min at room temperature and images were acquired using the UVP ChemiDoc-It™2 Imager and UVP software.

### 2.7. Determination of the Buoyant Properties of EVs

MuVs and lmEVs were resuspended in 500 µL of 0.25 M sucrose in 10 mM Tris, pH 7.4. and carefully loaded on a iodixanol/sucrose gradient as previously described [45]. Briefly, 5% *w/v*, 10% *w/v*, 20% *w/v*, and 40% *w/v* iodixanol solutions were prepared by diluting volumes of a stock solution of OptiPrep™ solution (60% *w/v* in water) with 0.25 M sucrose/10 mM Tris, pH 7.4. The gradient was then formed by adding 3 mL of the 40% iodixanol solution to a polycarbonate tube (Beckman Coulter) using a Pasteur pipette followed by the careful layering of the 20% *w/v* (3 mL), 10% *w/v* (3 mL) and 5% *w/v* (2.5 mL) solutions, respectively. The samples were then centrifuged at 100,000 g for 21 h at 4 °C. Twelve 1 mL fractions were collected from top to bottom and transferred to new tubes. Each fraction was then washed in 9 mL PBS and centrifuged at 100,000 g for 70 min at 4 °C. The resulting pellets were resuspended in 30 µL NuPage, loaded on 4–12% NuPAGE^®^ Bis-Tris gel, electrophoresed and transferred onto PVDF as described above. The membranes were probed for CD63 (MuVs marker; 10628D, Invitrogen, 1:1000), Annexin A1 (microparticle marker, A305-234A, Bethyl Laboratories, 1:1000) or ARF6 (microparticle marker, A235-238A, Bethyl Laboratories, 1:1000). The iodixanol density of each fraction was determined at 340 nm as previously described [45].

### 2.8. Culture of Human iPSC-Derived Motor Neurons (hMN)

Human iPSC-derived motor neuron progenitors were generated following the protocol as described [45,47] and characterized by our partner [48]. Progenitors were then differentiated into motor neurons. Briefly, µ-Slide 8-well tissue culture treated (Ibidi^®^) or 96-Well Black/Clear Bottom Plate (Thermo Fischer) were coated with 20 µg·mL^−1^ poly-L-ornithine overnight. After an initial wash with H_2_O, followed by PBS, each well was coated with 5µg·mL^−1^ laminin. After 24 h, human neuron progenitors were then plated and maintained in N2B27 media containing Neurobasal, DMEM/F12-Glutamax, N2 (1×), B27 (1×), Pen/Strep 1%, 0.25 mM 2-mercaptoethanol and 0.1% Glutamax^®^ and supplemented with 100 nM Rock Inhibitor (RI), 100 nM Retinoic Acid (RA), 500 nM SAG, 100 nM DAPT, 10 ng·mL^−1^ BDNF and 2.5 µg·mL^−1^ laminin. For the 8-well Ibidi slides for immunostaining, 6000 progenitors/well were plated, for the 96 Well plate for the uptake experiments 10,000 progenitor/well were plated. Two days later, the media was renewed with N2B27 media containing 200 nM RA, 1 µM SAG, 200 nM DAPT, 20 ng·mL^−1^ BDNF. After 3 days, the culture media was renewed with N2B27 media containing 200 nM RA, 200 nM DAPT, 20 ng·mL^−1^ BDNF and 20 ng·mL^−1^ GDNF. Two days later, the media was renewed with N2B27 media containing 20 ngmL^−1^ BDNF and 20 ng·mL^−1^ GDNF. At this point, progenitors were fully differentiated into motor neurons and ready for functional studies involving extracellular vesicles.

### 2.9. Culture of Astrocytes

Healthy mature human astrocytes (Gibco™) derived from human brain tissue were purchased from Thermo Fisher Scientific (N7805200) and cultured according to the manufacturer’s instructions. Tissue culture dishes or slides were coated with Geltrex™ Basement Membrane Matrix™ (prepared in DMEM) and incubated at 37 °C for 1 h. Prior to seeding cells, the dishes or slides were left at room temperature for 1 h followed by a single wash with DPBS containing calcium and magnesium. The cells were cultured at 37 °C, 5% CO_2._, in Gibco Astrocyte Medium comprising Dulbecco’s Modified Eagle’s Medium (DMEM), N-2 Supplement, and One Shot Fetal Bovine Serum (FBS).

Similarly, rat primary cortical astrocytes (N7745100, Gibco™, Thermo Fisher Scientific) were purchased and cultured according to the manufacturer’s instructions and grown in Dulbecco’s Modified Eagle’s Medium (DMEM; 85%) and Fetal Bovine Serum (FBS; 15%) at 37 °C, 5% CO_2_. Rat astrocytes were used for quantification of KI67 and GFAP immunostaining and to assess oxidative stress.

For immunostaining, 20,000 cells/well were plated on 8-well Ibidi slides. For the uptake experiments, 10,000 cells/well were plated on 96-well plates. For Western blot analysis, 200,000 cells per 10 cm Petri dish were plated.

### 2.10. Uptake of MuVs and lmEVs by iPSc Motor Neurons, Differentiated Myotubes, and Astrocytes

The uptake of MuVs and lmEVs was measured by PKH26 labelling as previously described [45]. Briefly, 100 μL of EV (MuVs or lmEVs) suspension was prepared and 100 μL of diluent C added. This was followed by the addition of 100 μL of 8 µM PKH26 and incubation of the mixture for 5 min. Thereafter, samples were washed thrice in PBS using an Amicon^®^ 100 K MWCO column at 15,000× *g* for 10 min at 4 °C. Labelled MuVs and lmEVs (85 ng of lipids) were then added to human iPSC MNs, astrocytes, or myotubes (10,000 cells per well per cell type), and incubated for 6 h at 5% CO_2_, 37 °C. Thereafter, the wells were gently washed 2 times with PBS, fixed with 4% paraformaldehyde (PAF) and the fluorescence from wells measured with a Biotek microplate reader using 485 and 535 nm as excitation and emission filters, respectively. Representative images were acquired for each cell type and EV type.

### 2.11. Protein Extraction from Treated Astrocytes

Muscle EVs (MuVs or lmEVs) were added to the culture medium of primary human astrocytes. At 72 h after treatment, treated and untreated astrocytes were washed with PBS. Cells were scraped with ice-cold RIPA buffer containing protease cocktail inhibitors, centrifuged at 10,000 g for 10 min at 4 °C. Protein quantification was performed using BCA and 25 µg of protein was separated on a 4–12% polyacrylamide Bis Tris Gel, transferred onto PVDF and blotted as described above. The membrane was probed with GFAP (marker for astroglial activation, PA1-10019, Invitrogen, 1:500), PCNA (marker for proliferation, PA5-16797, Thermo Fischer, 1:500), GAPDH (loading control, DSHB, 1:100) as well as anti-rabbit or anti-goat secondary antibodies conjugated with HRP (Thermo Fisher Scientific 1:10,000 and 1:8000, respectively) overnight. Thereafter, the membrane was incubated with Amersham ECL prime Western blotting detection reagent and images acquired using the UVP ChemiDoc-It™2 Imager and UVP software.

### 2.12. Immunolabeling

All images were acquired using an Olympus IX70 equipped with a Photomatics CoolSNAP™ HQ camera, and images were analyzed using Fiji software.

#### 2.12.1. For MN

After 72 h of treatment, MN were fixed using 4% formaldehyde, permeabilized, blocked and stained for Tuj1, Islet 1/2 as described [44]. Fifteen non-overlapping images were acquired across wells using an Olympus UPlan FI 10x/0.30 Ph1 objective. The MN death, neurite length, and branching were assessed.

#### 2.12.2. For Astrocytes

After 72 h of treatment with MuVs or lmEVs, astrocytes were fixed with 4% paraformaldehyde for 15 min. The slides were washed twice with PBS and blocked for 1 h with blocking buffer (20% FBS, 0.5% Tween 20, 0.1% Triton-X and 5% BSA). Ki-67 (Thermo Fischer, 1:200) or GFAP (Thermo Fischer, 1:200) primary antibody diluted in blocking buffer was added to the slides and incubated overnight at 4 °C. The slides were washed thrice with PBS and incubated with the secondary antibody (Goat anti-rabbit Alexa fluor 488 or 594; 1:400 dilution) for 90 min at room temperature. The slides were then washed thrice with PBS, and counterstained with 1 μg·mL^−1^ DAPI for 1 min. Anti-fading medium fluoromount was then added to each well. To assess the percentage of nuclei positive for Ki67, 15 non-overlapping images were acquired across wells using an Olympus LCPlan FI 401×/0.60 Ph2 objective. To assess GFAP staining, 15 non-overlapping images were acquired across wells using an Olympus UPlan FI 101×/0.30 Ph1 objective.

#### 2.12.3. For Myotubes

After 72 h of treatment, myotubes were fixed using 4% formaldehyde. The cells were permeabilized, blocked and stained for MF20 as described [49]. To assess the loss of myotubes, 15 non-overlapping images were acquired across wells using an Olympus UPlan FI 101×/0.30 Ph1 objective.

### 2.13. Quantification of Reactive Oxygen Species (ROS) Production by Astrocytes after Treatment with Muscle EVs

Astrocytes were plated in black 96-well flat bottom plates (Thermo Fisher Scientific Inc.). Post seeding and differentiation/adherence, cells were washed twice with PBS and preincubated with 50 µM 2′,7′-dichlorofluorescin diacetate (DCFH-DA) (Invitrogen™). Astrocytes were treated with MuVs or lmEVs (ALS: *n* = 3, healthy: *n* = 3, 85 ng of lipids). As a positive control, 100 µM H_2_O_2_ was added to the cells; as a negative control, 500 µM NAC (glutathione precursor and ROS scavenger) was added to the cells. Fluorescence was measured with a Biotek Multi-Mode Microplate Reader using 485 nm and 520 nm as excitation and emission filters, respectively.

### 2.14. Statistical Analysis

All values are presented as means ± SD. Sample number *n* indicates the number of subjects analyzed in each experiment. Mann–Whitney analysis was performed to compare the lipid concentration between MuVs and lmEVs. Two-way ANOVA followed by Šídák’s multiple comparisons test was performed to evaluate the uptake of MuVs and lmEVs by different cell types, the effects of MuVs and lmEVs on MN death, neurite length, the number of neurite branching points, the proportion of different astrocyte shapes, percentage of Ki67 positive astrocytes, GFAP expression level, differences in astrocyte shape and soma size, ROS production in astrocytes, myotubes death and ROS production in myotubes. A Kolmogorov–Smirnov test was used to compare the distribution of neurite branching in ALS and healthy MuV-treated MN, and in ALS and healthy lmEV-treated MN. Differences were considered to be statistically different at *p* < 0.05.

## 3. Results

### 3.1. Characterization of the EVs Secreted by Human Myotubes

**Figure 1:**
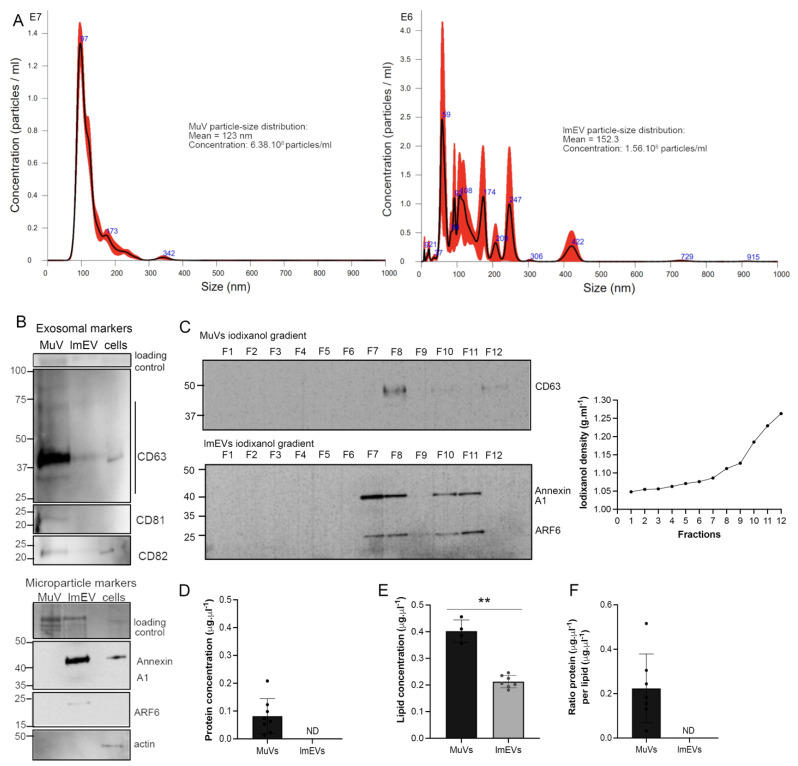
MuVs and lmEVs present different markers and have different buoyant properties. (**A**) histograms showing the MuV and lmEV particle-size distributions (representative sample, from ALS EVs). (**B**) Representative Western blots showing the detection of CD63, CD81, CD82, AnnexinA1, ARF6, and actin, in MuVs (line1), lmEVs (line 2) and cells (line 3). Exosomal markers were enriched in MuVs and at relatively low or undetectable levels in lmEVs (EVs were extracted from the same cell culture medium for both exosomal and microparticle markers). Protein loaded on the gel is also shown, as loading control. Cellular contamination was not observed as neither of the vesicle fractions were positive for alpha-skeletal actin. (**C**) Vesicle extracts loaded on iodixanol gradients. MuVs presented classic exosomal buoyant properties while the buoyant range of lmEVs extended to a higher iodixanol density. Top panel: representative Western blot showing detection of CD63 for the MuVs at a density of 1.112 g·mL^−1^; bottom panel: representative Western blot (EVs extracted from the same cell culture medium as top panel) showing detection of Annexin A1 and ARF6 for the lmEVs at a density of 1.086–1.112 g·mL^−1^ and 1.185–1.230 g·mL^−1^. Right panel: iodixanol/sucrose density across the 12 fractions; gradient range from 1.048 to 1.263 g·mL^−1^. (**D**) Protein concentration in MuVs samples. Protein quantification was below detection sensitivity for lmEVs (ND: not detected). (**E**) Lipid quantification in MuVs and lmEVs. **, *p* < 0.01. (**F**) Ratio of protein per lipid in MuVs and lmEVs. The protein/lipid ratio could not be determined for lmEVs as the protein quantity was below detection sensitivity.

Several markers were used to distinguish exosomal from non-exosomal properties of EVs: tetraspanins CD63, CD81, and CD82, are typical of exosomes [9], while Annexin A1 is a specific marker of microvesicles that are shed from the plasma membrane, and ADP-ribosylation factor 6 (ARF6) regulates abscission and shedding of microvesicles [50,51]. MuVs were enriched in tetraspanins, while lmEVs had relatively low or non-detectable levels of tetraspanins and were positive for Annexin A1 and ARF6 (Figure 1A). As a test for cellular contamination, alpha- skeletal actin was observed to be absent from all vesicle fractions. MuVs and lmEVs presented different buoyant properties, with the MuVs being detected at a density of 1.112 g·mL^−1^, and the lmEVs, which may be composed of a heterogeneous vesicle population with a wider range of densities, being detected from 1.086 to 1.230 g·mL^−1^ (Figure 1B). Protein concentration could be measured in MuVs and was at 0.081 μg·μl^−1^, while the protein concentration of lmEVs was below the sensitivity of detection (Figure 1C), whereas the lipid content could be measured in both MuVs and lmEVs, with concentrations of 0.402 ± 0.042 μg·μL^−1^ and 0.213 ± 0.023 μg·μL^−1^, respectively (Figure 1D). The ratio of protein per lipid for MuVs was 0.203 ± 0.155, similar to that previously described [46].

In summary, myotubes secreted significantly more MuVs than lmEVs, as assessed by protein and lipid concentrations; lmEVs extended to a higher range of densities and had lower protein content than MuVs.

### 3.2. MuVs Are Preferentially Absorbed by Motor Neurons

**Figure 2:**
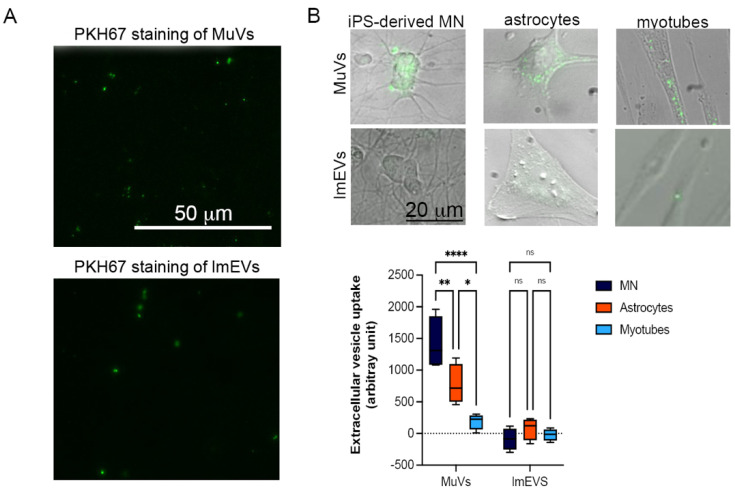
Uptake of MuVs or lmEVs by motor neurons, astrocytes, and myotubes. (**A**) MuVs and lmEVs labelled with PKH67 (droplet of vesicle preparations under fluorescence microscope), Bar = 50 μm. (**B**) A total of 85 ng of vesicular lipids were added to 10,000 cells. Top panel: representative images of MuV and lmEV uptake by hiPSC-derived motor neurons, astrocytes and myotubes. Vesicles were labeled with PKH67 (green). Bar = 25 μm. Bottom panel: the uptake of MuVs or lmEVs was assessed in healthy iPSC-derived motor neurons (MN), astrocytes, and myotubes (*n* = 4 per treatment, per cell line). Uptake of MuVs by MN was greater than by astrocytes or myotubes. Two-way ANOVA followed by Šídák’s multiple comparisons test. *, **, ****, *p* < 0.05, *p* < 0.01, *p* < 0.0001, respectively, ns: non-significant.

To compare the capacity of MuVs and lmEVs to be absorbed by different cell types, the same amount of vesicular lipids (85 ng/well) were loaded into each well, with each well containing the same number of cells (10,000 cells per well), over a 6 h period. Compared to astrocytes or myotubes, motor neurons absorbed a significantly greater amount of MuVs (Figure 2). The myotubes were the less receptive cells for MuVs among the three cell types tested. On the other hand, lmEVs were not detected in any recipient cells.

### 3.3. The MuVs, but Not lmEVs, of ALS Subjects Have an Effect on MN and Myotube Survival

**Figure 3:**
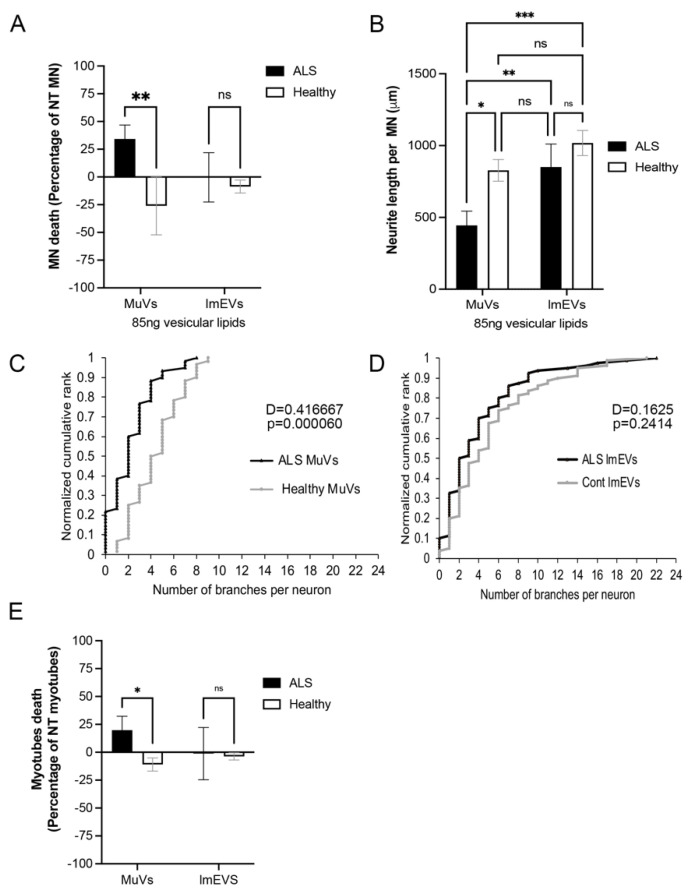
MuVs, but not lmEVs, have an effect on MN survival, neurite length, and neurite branching, and on myotube survival. iPSC-MN or myotubes were treated with either MuVs or lmEVs secreted from the muscle cells of either ALS subjects or healthy subjects. (**A**) Motor neuron death as a percentage reduction in MN numbers compared to untreated MN (*n* = 3/4 per group). **, *p* < 0.01. (**B**) Measurements of neurite lengths per MN. *, **, ***, *p* < 0.05, *p* < 0.01 and *p* < 0.001 respectively, ns: non-significant. (**C**) Neurites of iPSC-MN cells had fewer neurite branch-points following treatment with ALS MuVs compared to treatment with healthy MuVs, while (**D**) no difference was observed in the effects of ALS lmEVs compared to healthy lmEVs; the D statistic and *p*-values are indicated for two-sample Kolmogorov–Smirnov tests (ALS vs. healthy MuVs, *p* = 0.00006; ALS vs. healthy lmEVs, *p* = 0.24). (**E**) Myotube death as a percentage reduction in myotube numbers compared to untreated myotubes (*n* = 3 per group). *, *p* < 0.05; ns—non-significant.

We then compared the impact of MuVs and lmEVs on MN survival, neurite length, and neurite branching, over a 72 h period. Similarly to our previous observations [44], MuVs secreted by the muscle cells of ALS subjects (ALS MuVs) had a negative effect on MN survival (Figure 3A), neurite length (Figure 3B), and the number of neurite branches (Figure 3C), compared to those secreted by the muscle cells of healthy subjects (Healthy MuVs). MuVs of healthy subjects had a protective effect on MN survival (Figure 3A). However, neither ALS lmEVs nor Healthy lmEVs had an effect on MN (Figure 3A,B,D).

Similarly to our previous observations [44], ALS MuVs (85 ng of lipids) added to the culture medium of healthy human myotubes for 72 h induced muscle death. However, no effect was observed when adding the same amount of ALS or Healthy lmEVs (Figure 3E).

### 3.4. Effects of MuVs or lmEVs on Astrocytes

**Figure 4:**
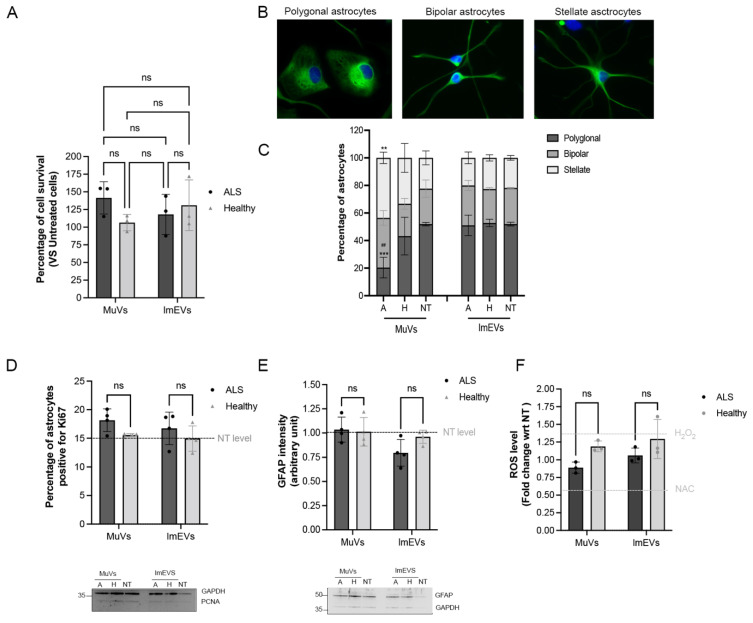
Effects of MuVs or lmEVs on astrocytes. (**A**) Quantification of astrocyte survival following treatment with EVs secreted by the myotubes of ALS or healthy subjects. (**B**) Representative images of astrocyte shapes. Green: GFAP staining, blue: DAPI. (**C**) Distributions of astrocyte shapes when treated or not with MuVs (NT-M = non-treated, H-M = treated with Healthy MuVs, A-M = treated with ALS MuVs) or with lmEVs (NT-L = non-treated, H-L = treated with Healthy lmEVs, A-L = treated with ALS lmEVs), (*n* = 4 per treatment). **, *p* < 0.01, ***, *p* < 0.001, significantly different from NT, ##, *p* < 0.01, significantly different from Healthy MuV values, ns: non-significant. Plots are shown for (**D**) percentage of Ki67 positive astrocytes, (**E**) measurement of GFAP intensity in astrocytes, and (**F**) quantification of ROS production in rat astrocytes, following treatment with MuVs (ALS or Healthy) or lmEVs (ALS or Healthy) (*n* = 3–4 per treatment). Dotted lines indicate levels for untreated cells or in (**D**) for H_2_O_2_ positive control and NAC (N-acetyl cysteine) negative control. Lower panels: representative Western blots showing (**D**) the low expression level of PCNA in astrocytes, and (**E**) the constant expression level of GFAP in astrocytes, regardless of treatment; ns—non-significant.

Cell death effects were not observed on astrocytes at 72 h (Figure 4A). We then checked whether astrocytes became activated, either proliferating or increased in cellular stress when treated with different types of vesicles over the same time period. Compared with Healthy MuVs, the treatment of astrocytes with ALS MuVs significantly increased the proportion of stellate astrocytes (with a corresponding reduction in the proportion of polygonal astrocytes), suggesting that ALS MuVs increase the tendency of astrocytes to branch (Figure 4B,C). In contrast, assays of astrocyte proliferation and activation (Ki67, Figure 4D; GFAP expression level, Figure 4E), or levels of oxidative stress (Figure 4F), all showed no observed effect of treatment with any muscle EVs (neither MuV nor lmEV, neither ALS nor Healthy) compared to untreated astrocytes. In summary, these data suggest that ALS MuVs have a mild activation effect on astrocytes, while no obvious changes were observed when astrocytes were treated with healthy MuVs, ALS lmEVs, or healthy lmEVs.

## 4. Discussion

Eukaryotic cells are reported to release extracellular vesicles under conditions of both normal and pathological physiology, with the function(s) of the released vesicles dependent on the content of the cargo and the donor cell under investigation [52]. EVs participate in cell-to-cell communication that takes place between and within similar or different cell types, and they are implicated in neurodegenerative diseases where they mediate the transport and spread of misfolded proteins, lipids, and nucleic acids [53]. In this study, we report that EVs of skeletal muscle cells extracted at g of 20,000 (lmEVs) or 100,000 (MuVs) exhibit marked differences in their composition and in their uptake by and effects on recipient motor neuron, myotube, and astrocyte cells.

In accordance with our previous studies [44,45], MuVs were enriched in tetraspanins that could be involved in receptor-mediated endocytosis and cell-to cell signaling [13], negative for ectosome markers, and had a protein:lipid ratio similar to what is reported for exosomes in the literature. On the other hand, lmEVs were positive for Annexin A1 and ARF6-protein markers associated with vesicles that bud directly from the plasma membrane [50,51] and their protein content was below detection limit. The density gradient centrifugation confirmed that the densities of lmEVs occupied a greater range and extended higher, floating between 1.086 and 1.230 g·mL^−1^, than MuVs, which were found at 1.112 g·mL^−1^. Ectosomes have previously been reported to have a buoyant density of 1.14–1.20 g·mL^−1^ [54], although the methods of centrifugation and the type of cell under investigation could influence the size and density of secreted vesicles. Per sample volume, the MuV fraction contained a significantly greater amount of total lipid than the lmEV fraction, suggesting that MuVs are secreted to a greater extent compared to lmEVs. Le Bihan et al. [13] reported a significant difference (65%) in protein content, with MuVs being enriched in plasma membrane proteins (integrins and tetraspanins). Similarly, Keerthikumar et al. [54], using SH-SY5Y neuroblastoma cells confirmed that exosomes and ectosomes have markedly different protein profiles, with exosomes containing more oncogenic proteins.

Following release from donor cells, EVs are taken up by recipient cells via mechanisms that include plasma or endosomal membrane fusion, endocytosis, phagocytosis and micropinocytosis [9], allowing EVs to transfer proteins, lipids, and nucleic acids. EV uptake is an active process occurring as early as 15 min after addition to the medium, and uptake will be variable depending on the types of the source and recipient cells [55]. In the present study, MuVs were preferentially taken up by motor neurons, with a ratio of 2.5 and 10 times greater uptake compared to astrocytes and myotubes over a 6 h period, respectively. However, lmEVs were barely taken up by any of the three cell types. These findings are consistent with observations on the internalization kinetics of exosomes and ectosomes into differentiated myotubes, in which internalized exosomes were readily detected after 5 h, but ectosomes required 10 h for detection [13]. Taken together, these data suggest differences between the two types of EVs, in terms of cell type tropism and/or internalization kinetics. The specificity and stochasticity of EV uptake and the molecular mechanisms that underlie them still need to be investigated [55,56].

Similarly to our previous study [44], we confirmed that ALS MuVs affect motor neuron survival, causing a shortening of neurite lengths and a decrease in neurite branching, while Healthy MuVs appear to present a protective effect on human MN. This latter observation is consistent with an in vitro murine model where exosomes derived from C_2_C_12_ myotubes promoted neuron survival and neurite outgrowth when applied to NSC34 cells [29]. In contrast, neither ALS lmEVs nor Healthy lmEVs had an effect on motor neuron survival.

Other data support the uptake of CD63-positive EVs by motor neurons. MuVs are enriched in CD63, and when MuVs were coated with anti-CD63, we observed that the uptake by MN was abolished [44]. CD63-positive EVs were previously observed to bind to neuronal and glial cells [57], and CD63-enriched exosomes have been associated with the transsynaptic spread of tau pathology in AD [58], and with increased dissemination of infectious viral components [59]. It is plausible that the uptake of CD63 enriched vesicles by MN could participate to cell–cell communication and/or propagation of toxic elements.

Following the release of vesicles into the extracellular space, they can then target other cell types. Notably, skeletal muscle derived exosomes transfer toxic materials to neighboring cells, causing homeostatic alterations [31] that were suggested to target certain tissues, including the brain. For example, myotube-derived exosomes from atrophied muscles are enriched in microRNAs, especially in miR-29b-3p, which can be efficiently transferred to neuronal cells, inhibiting neuronal differentiation, and possibly affecting the integrity of the neuromuscular junction (NMJ) [60]. An in vivo study by Banks et al. investigating uptake of exosomes of different cell origins in the context of exosome–brain interactions revealed that all tested exosomes crossed the brain–blood barrier (BBB), albeit at differing rates and with even distribution throughout the brain including the olfactory bulb, cortex, and cerebellum [61].

The absorption by astrocytes of exosomes derived from neurons, monocytes, and glial cells, as well as stem cells, has been investigated in some depth [62]. In the present study, we observed that MuVs were moderately absorbed by astrocytes. Morphological indication of human astrocyte activation [63,64] was observed in the form of increased branching. However, this was not accompanied by increased proliferation of astrocytes, nor by increased oxidative stress nor astrocyte death. In agreement with these data, a previous study showed only minimal uptake of EVs by astrocytes [65].

In conclusion, since toxicity towards motor neurons was observed only for ALS MuVs and not for Healthy MuVs, ALS lmEVs, or Healthy lmEVs, these findings support a role for muscle EVs of specifically exosomal type in the transmission of neurotoxic elements in ALS pathology. Further study is needed to determine whether this finding is specific to EVs of muscle origin or can be extended to include EVs of any origin. The observation that the absorbance of MuVs is 10 and 2.5 times greater in motor neurons than in myotubes and astrocytes, respectively, may be of interest when considering the cell type specificity of ALS pathology.

## Figures and Tables

**Table 1:** Table showing the gender, age and ALS mutations of the subjects. nmi: no ALS mutations identified.

Group	Gender	Age	ALS Mutation	EVs Used in Treatment of		
				MNs	Myotubes	Astrocytes
ALS	F	50–59	*C9orf72*	×		×
ALS	M	50–59	nmi	×		×
ALS	M	50–59	*C9orf72*		×	
ALS	M	40–49	nmi	×		
ALS	M	60–69	nmi	×		×
ALS	M	60–69	nmi		×	×
ALS	M	70–79	nmi		×	×
Healthy	F	60–69	-			×
Healthy	F	50–59	-		×	
Healthy	M	20–29	-			×
Healthy	M	70–79	-	×		
Healthy	M	30–39	-	×	×	
Healthy	M	20–29	-	×		×
Healthy	M	50–59	-	×	×	×

## Data Availability

This study did not involve the generation of large public datasets. The data presented are available from the corresponding author upon request.
